# Adherence to inhalers and comorbidities in COPD patients. A cross-sectional primary care study from Greece

**DOI:** 10.1186/s12890-020-01296-3

**Published:** 2020-09-25

**Authors:** Despo Ierodiakonou, Dimitra Sifaki-Pistolla, Maria Kampouraki, Ioannis Poulorinakis, Polyvios Papadokostakis, Ioannis Gialamas, Polyxeni Athanasiou, Vasiliki Bempi, Irene Lampraki, Ioanna Tsiligianni, On behalf of the Greek UNLOCK group, Maria Kampouraki, Despo Ierodiakonou, Ioanna Tsiligianni, Eleftheria Lintovoi, Dimitris Karanassos, Polyvios Papadokostakis, Ioannis Poulorinakis, Kyriakos Maltezis, Maria Chorti, Evangelos Petrovitsos, Sofia Dimopoulou, Sam Hamind, Ioannis Gialamas, Polyxeni Athanasiou, Vasiliki Bempi, Irene Lampraki

**Affiliations:** 1 Health Planning Unit. Department of Social Medicine, Faculty of Medicine, University of Crete, Voutes Campus, Heraklion, GR-71003 Crete, Greece; 2 Department of Public Health, Heraklion University Hospital, Heraklion, Crete, Greece; 3 Clinic of Social and Family Medicine, Faculty of Medicine, University of Crete, Crete, Greece; 4 Primary Care Practice, Health Center of Moires, Heraklion, Crete, Greece; 5 Primary Care Practice, Health Center of Agia Varvara, Heraklion, Crete, Greece; 6 Primary Care Practice, Garipa, Heraklion, Crete, Greece; 7 Primary Care Practice, Health Center of Sitia, Sitia General Hospital, Lasithi, Crete, Greece

**Keywords:** COPD, Adherence, Comorbidities, Exacerbations, Health status, GOLD2019

## Abstract

**Background:**

Comorbidities and adherence to inhaled therapy appears to have a major impact on treatment goals, health status and disease control in chronic obstructive pulmonary disease (COPD). Aim of the study was to assess levels of adherence to inhalers, comorbidities and associations with COPD outcomes in patients residing in rural and semi-urban areas of Greece.

**Methods:**

Two hundred fifty-seven COPD patients were enrolled from primary health care in 2015–2016. Physicians used structured interviews and questionnaires to assess quality of life and disease status. Patients were classified into groups according to GOLD 2019 guidelines (based on CAT and mMRC). Adherence to inhalers was measured with the Test of Adherence to Inhalers (TAI). Multivariate linear and logistics regression models were used to assess associations between comorbidities and adherence to inhalers with COPD outcomes, including CAT and mMRC scores, exacerbations and GOLD A-D status.

**Results:**

74.1% of COPD patients reported poor adherence, while most of them were characterized as deliberate non-compliers (69.5%). 77.1% had ≥2 comorbidities, with overweight/obese (82.2%), hypertension (72.9%) and diabetes mellitus (58%) being the most prevalent. In multivariate analysis, COPD outcomes having significant associations with poor adherence included worse health status [OR (95% CI) 4.86 (1.61–14.69) and 2.93 (1.51–5.71) based on CAT and mMRC, respectively], having ≥2 exacerbations in the past year [4.68 (1.51–4.44)], and disease status e.g., be in groups C or D [3.13 (1.49–8.53) and 3.35 (1.24–9.09) based on CAT and mMRC, respectively). Subjects with gastroesophageal reflux showed better inhaler adherence [OR (95% CI) 0.17 (0.6–0.45)], but none of the comorbid conditions was associated with COPD outcomes after adjustments.

**Conclusions:**

Poor adherence to inhalers and comorbidities are both prevalent in COPD patients of primary care residing in rural/semi-urban areas of Greece, with adherence influencing COPD outcomes. Raising awareness of patients and physicians on the importance of comorbidities control and inhaler adherence may lead to interventions and improve outcomes.

## Background

Chronic obstructive pulmonary disease (COPD) is a challenging public health problem [1, 2] and relatively under-diagnosed in many rural and semi-urban regions worldwide [3–5]. Effective control of COPD is crucial for enhancing quality of life (QoL) in COPD patients, diminishing exacerbations, hospitalizations and mortality [6–8]. Progressive introduction of pharmacologic or nonpharmacologic interventions are often the key components towards coping with COPD [9]. Nevertheless, effectiveness of COPD treatment also lies on patient adherence to intended treatment [10]. Adherence to inhalers in COPD patients is found to be relatively poor with non-adherence rates ranging between 50 and 80% [11–14]. In a real-life cross-sectional non-interventional study in Latin America (LASSYC), only 50% of the patients had good adherence [12]. A recent review of findings supports that patients with good adherence to inhaled medication tends to improve course of exacerbations and decreases risk of mortality [15]. Similarly in Greece, a nationwide COPD study showed that moderate to poor adherence approximated 50% [16] and in another study patient-reported non-adherence led to 11.5% more exacerbations and 14.1% more hospitalizations per year [16], with economic impact as well [17].

Possible factors influencing non-adherence rates are not fully understood, but recent studies have revealed that rural and remote areas, regions undergoing financial or social crisis, and older patients with multiple morbidities tend to present much higher non-adherence rates [3–5]. More specifically, healthcare resources in Greece are unevenly distributed across the country and even though more than a quarter of the population resides in rural areas, rural GP practices in Greece report shortages of GPs, nursing staff and equipment [18] which may have serious impact on COPD management e.g., patient training and spirometry. In addition, a study from Greece has emphasized the significant financial impact on COPD patients’ adherence to therapy in rural areas during the austerity period [4]. The above highlight the importance of assessing patient adherence in primary care settings of rural areas in Greece and its potential impact on COPD outcomes.

The Global Initiative for Chronic Obstructive Lung Disease (GOLD) in the 2019 guidelines includes assessment of adherence in the follow up, pharmacological management recommendations, and also underlines the importance of managing the comorbidities in subjects suffering from COPD [19]. Approximately, 86 to 98% of individuals with COPD have at least one comorbid condition and the mean number of comorbidities per subject is 1.2 to 4 [20, 21]. Attention given on presence of comorbidities in COPD is mainly attributed to evidence suggesting that COPD patients with co-existing pathologies may have worse disease prognosis, QoL and mortality [19–24]. The German COPD and Systemic Consequences-Comorbidities Network (COSYCONET) was a large cohort study which showed that the recently modified GOLD categorization correlated with risk of important comorbidities, and underlined the importance of identifying comorbidities in COPD, particularly in non-responders to therapy who had high symptoms and/or exacerbation rate [25]. Although several observational studies evaluated comorbidities and adherence to inhalers in COPD patients in different countries, as well as their associations with COPD outcomes, data are lacking from primary care settings of rural areas in Greece which may be at higher risk.

## Methods

The present study explores in real-life the levels of adherence to inhalers in COPD patients residing in rural and semi-urban areas of Greece, their co-morbidities, and assesses associations of these two predictors with COPD outcomes.

This real-life study was conducted by the Greek group of an international collaboration between primary care researchers. The protocol of the UNLOCK (Uncovering and Noting Long-term Outcomes in COPD and asthma to enhance Knowledge) was published in 2010 [26].

### Study populations

We used a convenience sampling approach to select COPD patients served by primary care and living in rural and semi-urban areas across Greece. The study was approved by the local medical ethics committee of the University Hospital of Crete Greece (protocol number 7985) and the patients gave written informed consent. Detailed methods have been previously published [26–28]. Subjects were recruited by 53 facilities with participants ranging between 5 and 20 patients per area. The participation rate of subjects varied from 78 to 91%. In summary, 257 COPD patients, previously diagnosed by spirometry (e.g. a post-bronchodilator forced expiratory volume in 1 sec (FEV_1_)/forced vital capacity (FVC) ≤0.7 in patients with symptoms and/or history of smoking) [29] during an assessment by chest physicians, were enrolled between 2015 and 2016. Through structured interviews, the general practitioners collected cross-sectional information including demographic characteristics, medical history, lifestyle, COPD Assessment Test (CAT [30]) and Modified Medical Research Council Dyspnea Scale (mMRC [31, 32]) scores, annual number of exacerbations and hospitalisations. COPD patients were classified with ABCD grading system according to GOLD 2019 guidelines [19].

### Comorbidities

Comorbidities were provided by the primary care physicians based on known patient medical history and/or chronic medication used. Findings from the COPDGene study showed that a comorbidity count performs well to quantify comorbidity in a diverse population with COPD, compared to weighted score or weighted score based upon statistical selection procedure [33], therefore we used a comorbidity count (sum of comorbidities; range from 0 to 9) and grouped our COPD patients into those with at least two (≥2) comorbidities or less.

### Adherence to inhalers

Adherence to inhalers in COPD patients was assessed with the Test of Adherence to Inhalers (TAI; http://www.taitest.com/) [34]. It is a 12-item questionnaire which is designed to assess difficulties in following treatment with inhalers in people with asthma or COPD. It consists of 10 items addressing the patient, and 2 additional items which the practitioner can use to identify patients with low adherence. The 10-item TAI is used by healthcare professionals to assess only adherence and degree of adherence, and they may use the 12-item TAI to assess type of non-compliance. Scoring of each of the 10-item ranges from 1: worst compliance to 5: best compliance, providing a total sum score of between 10 (minimum) and 50 (maximum), which can determine the degree of adherence: good (50 points), intermediate (46 to 49 points) or poor (≤ 45 points). Items for healthcare professionals are scored with 1 or 2 points (poor or good knowledge of the regimen and/or inhalation technique) and may offer insights on patient’s type or pattern of non-compliance as follows: sporadic, deliberate or unconscious [34, 35]. A single patient may have more than one type or pattern of non-compliance and a patient with good adherence may be an unconscious non-complier.

### Statistical analysis

Baseline characteristics are presented as count (n) and percentage (%), or mean and standard deviation (SD). If the distribution of continuous data was not normal, then median (interquartile range (IQR)) was used. Univariate analysis was performed using Chi-square or Fisher’s exact tests for categorical outcomes, and Student’s t-test or Mann-Whitney U test for continuous outcomes with normal or not distribution, respectively. Moreover, multivariate logistic regression models were developed to test associations of comorbidities and poor adherence to inhalers (intermediate and good adherence pooled as reference group) with COPD outcomes, adjusting for age, gender, smoking status and occupational status. All tests were two-tailed with significance level at 5%, and were performed in the IBM-SPSS Statistics software (version 23).

## Results

The main population characteristics and disease status in COPD patients for the total sample as well for inhaler adherence groups are presented in Table 1. Mean (SD) age was 65 (12.3) years with male predominance (*n* = 204 (79.4%)). According to CAT (≥10) and mMRC (≥2) tools, uncontrolled symptoms/poor health status was found in 224 (91.1%) and 154 (60.6%), respectively. ≥2 exacerbations were seen in 77 (37.2%) subjects, with GOLD group B being the largest followed by groups D, A and C.

**Table 1:** Population characteristics and health status of 257 COPD patients of rural/semi-urban areas of Greece

Characteristics and health status	Total Group ***N*** = 257	Good-Intermediate adherence ***N*** = 59 (25.9%)	Poor adherence ***N*** = 169 (74.1%)	***P***-value*
**Age; mean (SD)**	65 (12.3)	69.3 (13.4)	64.7 (12.0)	0.004
**BMI; mean (SD)**	29 (5.3)	29.7 (6.1)	29.3 (5.0)	0.603
**Males; n (%)**	204 (79.4)	45 (76.3)	133 (78.7)	0.837
**Occupational status; n (%)**				
Employed	74 (33.3)	10 (20.8)	52 (33.5)	0.038
Unemployed/housewife	25 (11.3)	3 (6.3)	22 (14.2)	
Retired	123 (55.4)	35 (72.9)	81 (52.3)	
**Smoking status; n (%)**				
Current	143 (55.6)	25 (42.4)	98 (58)	0.120
Ex	83 (32.3)	25 (42.4)	50 (29.6)	
Never	31 (12.1)	9 (15.3)	21 (12.4)	
**Pack-years; median (IQR)**	40 (10)	40 (60)	40 (10)	0.003
**CAT score; mean (SD)**	17.2 (6.7)	17.5 (7.3)	17.2 (5.7)	0.170
**CAT score ≥ 10; n (%)**	224 (91.1)	45 (83.3)	155 (95.1)	0.015
**mMRC score; mean (SD)**	1.9 (1.2)	1.7 (1.4)	2 (1.06)	0.759
**mMRC score ≥ 2; n (%)**	154 (60.6)	28 (47.1)	111 (66.5)	0.013
**Number of exacerbations in the last 12 months**^**a**^**; median (IQR)**	1 (1)	1 (1)	1 (1)	0.001
**Exacerbations in the last 12 months; n (%)**				
**0**	55 (16.2)	16 (43.2)	37 (13.2)	
**1**	167 (49.3)	16 (43.2)	136 (48.6)	< 0.001
**≥ 2**	117 (34.5)	5 (13.5)	107 (38.2)	
**Number of hospitalisations in the last 12 months; median (IQR)**	0 (0)	0 (0)	0 (0)	0.913
**Hospitalised in the last 12 months; n (%)**	12 (5.2)	7 (15.2)	4 (2.1)	0.003
**GOLD 2019 – CAT; n (%)**				
**A**	11 (5.4)	4 (13.8)	6 (3.8)	
**B**	115 (56.4)	18 (62.1)	86 (54.4)	0.082
**C**	3 (1.5)	0 (0)	2 (1.3)	
**D**	75 (36.8)	7 (24.1)	64 (40.5)	
**GOLD 2019 – mMRC; n (%)**				
**A**	57 (27.5)	14 (46.7)	38 (23.6)	
**B**	71 (34.3)	9 (30.0)	56 (34.8)	0.055
**C**	20 (9.7)	1 (3.3)	16 (9.9)	
**D**	59 (28.5)	6 (20.0)	51 (31.7)	

*BMI* Body mass index, *IQR* Interquartile range, *GOLD 2019* Global Initiative for Obstructive Lung Disease 2019 Guidelines, *CAT* Chronic Obstructive Pulmonary Disease Assessment Test, *mMRC* Modified Medical Research Council Dyspnoea Scale

*Chi-square or Fisher’s exact test for categorical variables, Student’s T-test for continuous variables with normal distribution and Mann-Whitney U test for not normally distributed variables

^a^An exacerbation was defined based on GOLD as “an event in the natural course of the disease characterized by a change in the patient’s baseline dyspnea, cough, and/or sputum that is beyond normal day-to-day variations, is acute in onset, and may warrant a change in regular medication to a patient with underlying COPD”, and patients were specifically asked whether they underwent such a change in the past 12 months

Comorbidities of COPD subjects are presented in Table 2. Subject’s mean (SD) number of comorbidities was 2.3 (1.2) [min-max 0–6]. One hundred and eighty-nine (77.1%) COPD patients suffered from ≥2 comorbidities, but when overweight/obese was excluded from the comorbidity sum score this number dropped to 108 (42.5%) subjects. Cardiovascular risk factors were common including overweight/obese (n (%) 203 (82.2%)), hypertension (186 (72.9%)), hyperlipidaemia (63 (24.6%)) and type II diabetes mellitus (28 (11%)). Heart disease, osteoporosis and gastroesophageal reflux disease (GERD) were significantly associated with adherence to inhalers, however after adjustments for age sex, smoking, overweight/obese and occupational status, only association with GERD remained significant (OR (95% CI) 0.17 (0.6–0.45)), suggesting that subjects with GERD were more likely to comply with their inhalers.

**Table 2:** Comorbidities in COPD patients of rural/semi-urban areas of Greece

N (%)	Total Group ***N*** = 257	Good-Intermediate adherence ***N*** = 59 (25.9%)	Poor adherence ***N*** = 169 (74.1%)	***P***-value*
≥2 c**omorbidities**	189 (77.1)	43 (79.6)	129 (77.7)	0.915
**Overweight-obese (≥ 25 Kg/m**^**2**^**)**	203 (82.2)	43 (78.2)	142 (85.5)	0.284
**Type II Diabetes Mellitus**	28 (11.0)	9 (15.5)	17 (10.1)	0.375
**Hypertension**	186 (72.9)	39 (67.2)	127 (75.1)	0.317
**Dyslipidaemia**	63 (24.6)	17 (28.8)	41 (24.3)	0.605
**Heart Disease**	32 (12.5)	14 (23.7)	17 (10.1)	0.016
**Thyroid disorder**	19 (7.4)	7 (11.9)	11 (6.5)	0.259
**Osteoporosis**	7 (2.7)	6 (6.8)	2 (1.2)	0.040
**Depression**	23 (9)	6 (10.2)	16 (9.5)	0.999
**Gastro-oesophageal reflux**	30 (11.7)	20 (33.9)	9 (5.3)	< 0.001
**Cancer (any)**	3 (1.2)	2 (3.4)	1 (0.6)	0.165

*Chi-square or Fisher’s exact test

Table 3 shows the adherence to inhalers in COPD patients and type of non-compliance based on TAI. Good adherence was found in 43 (18.9%) subjects, intermediate adherence in 16 (7.0%) and poor adherence in 169 (74.1%). A single patient may have more than one type or pattern of non-compliance and a patient with good adherence may be an unconscious non-complier. One hundred and eighty-three (79.9%) subjects presented sporadic non-compliance, 169 (69.5%) deliberate non-compliance and 99 (41.8%) unconscious non-compliance. Table 4 shows type of non-compliance within levels of adherence in COPD patients. The vast majority of subjects with poor adherence showed sporadic (100%) and/or deliberate non-compliance (93%), with only 51% presenting with unconscious non-compliance; opposed to subjects with intermediate/good adherence of whom none showed deliberate non-compliance while only 16 and 11% presented with sporadic and unconscious non-compliance, respectively.

**Table 3:** Adherence to inhalers of COPD patients and overall type of non-compliance

	N (%)
**TAI-10 items**^**a**^	
Good adherence	43 (18.9)
Intermediate adherence	16 (7.0)
Poor adherence	169 (74.1)
**TAI-12 items**^**b**^	
Sporadic non-compliance	183 (79.9)
Deliberate non-compliance	169 (69.5)
Unconscious non-compliance	99 (41.8)

^a^mean (sd) TAI-10 score: 37.8 (7.9)

The 10-item TAI identifies the following levels of adherence [35]: Good adherence: compliant patient. Intermediate adherence: moderately compliant patient. Poor adherence: non-compliant patient. The definitions for the types of non-compliance that the 12-item TAI identifies are the following [35]: Sporadic non-compliance: patient who forgets to take their medication. Deliberate non-compliance: patient who does not take their medication because they do not want to. Unconscious non-compliance: patient who does not take their medication properly because they do not know the therapeutic regimen and how to use their inhaler device

^b^a single patient may have more than one type or pattern of non-compliance and a patient with good adherence may be an unconscious non-complier; TAI: Test of Adherence to Inhalers

**Table 4:** Type of non-compliance within level of adherence in COPD patients

TAI-12 items^**a**^	Sporadic non-compliers (***n*** = 183)	Deliberate non-compliers (***n*** = 169)	Unconscious non-compliers (***n*** = 99)
**Poor Adherence (*n*** **= 169)**	169 (100%)	158 (93.5%)	84 (51.5%)
**Good-Intermediate Adherence (*n*** **= 59)**	10 (16.9%)	0	7 (11.9%)

*TAI* Test of Adherence to Inhalers;

^a^a single patient may have more than one type or pattern of non-compliance and a patient with good adherence may be an unconscious non-complier; TAI: Test of Adherence to Inhalers

The proportion of patients with at least two comorbidities was significantly associated with CAT-based GOLD status (Fisher’s exact test *p* = 0.034); 45.5% in GOLD group A, 75.5% in GOLD group B, 33.3% in GOLD group C and 78.7% in GOLD group D patients. Univariate associations of comorbidities with COPD outcomes are shown in Table 5. Subjects suffering from hypertension and type II diabetes mellitus were more likely to be in group C and D compared to subjects without hypertension or diabetes. Hypertension was also significantly associated with ≥2 COPD exacerbations in the past year. Nevertheless, when we corrected for potential confounders, only associations of diabetes with GOLD 2019 status remained significant (Table 6). Other recorded chronic diseases (osteoporosis, gastro-oesophageal reflux disease, thyroid disease, depression, cancer) were not associated with COPD outcomes (data not shown).

**Table 5:** COPD outcomes according to main morbidities

	At least 2 comorbidities			Overweight-Obese			Hypertension			Type II Diabetes Mellitus			Dyslipidaemia			Heart Diseases		
COPD outcome	present	absent	***p***-value	present	absent	***p***-value	present	absent	***p***-value	present	absent	***p***-value	present	absent	***p***-value	present	absent	***p***-value
**At least 2 exacerbations in the last 12 months**	58 (38)	19 (37)	0.999	61 (36)	16 (49)	0.239	63 (42)	14 (25)	**0.037**	11 (58)	66 (35)	0.081	13 (29)	64 (40)	0.259	12 (48)	65 (36)	0.332
**CAT ≥ 10**	13 (7)	8 (15)	0.095	19 (10)	3 (8)	0.776	164 (92)	58 (88)	0.436	26 (96)	197 (91)	0.484	57 (93)	166 (90)	0.613	30 (97)	193 (90)	0.326
**mMRC ≥ 2**	113 (61)	34 (61)	0.999	127 (64)	21 (48)	0.077	113 (61)	39 (57)	0.660	21 (75)	132 (59)	0.151	31 (50)	122 (64)	0.073	24 (75)	128 (58)	0.109
**GOLD 2019-C&D status** **CAT-based**	60 (40)	18 (37)	0.739	64 (38)	14 (45)	0.548	64 (43)	14 (26)	**0.023**	13 (62)	65 (36)	**0.031**	15 (33)	63 (40)	0.395	13 (52)	65 (36)	0.186
**GOLD 2019-C&D status** **mMRC-based**	60 (40)	19 (37)	0.869	63 (37)	16 (49)	0.245	65 (43)	14 (26)	**0.024**	13 (62)	66 (36)	**0.031**	15 (33)	63 (40)	0.395	13 (50)	66 (37)	0.200

Cell values are presented as n (% of COPD outcome within morbidity status) of subjects with COPD outcome in each comorbid condition group and *P*-values are given by Chi-square test. Bold faceted text indicates *P*-values< 0.05. *COPD* Chronic obstructive pulmonary disease, *GOLD 2019* Global Initiative for Obstructive Lung Disease 2019 Guidelines, *CAT* Chronic Obstructive Pulmonary Disease Assessment Test, *mMRC* Modified Medical Research Council Dyspnoea Scale

**Table 6:** Associations of diabetes and poor adherence to inhalers with COPD outcomes

	Diabetes^**a**^		Poor adherence^**a**^	
COPD outcome	OR (95% CI)	***P***-value	OR (95% CI)	***P***-value
**CAT ≥ 10**	0.49 (0.05–4.50)	0.531	**4.86 (1.61–14.69)**	**0.005**
**mMRC ≥ 2**	0.41 (0.15–1.21)	0.082	**2.93 (1.51–5.71)**	**0.002**
**≥ 2 Exacerbations**	0.47 (0.17–1.31)	0.151	**4.68 (1.51–4.44)**	**0.007**
**GOLD 2019 – C&D** **CAT based**	**2.83 (1.04–7.71)**	**0.041**^**⁋**^	**3.13 (1.49–8.53)**	**0.026**
**GOLD 2019 – C&D** **mMRC based**	**2.83 (1.04–7.72)**	**0.042**^**⁋**^	**3.35 (1.24–9.09)**	**0.017**

^a^Multivariate logistic regression models including both predictors adjusted for gender, age and smoking status. Bold faceted text indicates *P*-values< 0.05. ^⁋^ *P*-value> 0.05 when adding occupational status into the model. *OR* Odds ratio, *COPD* Chronic obstructive pulmonary disease, *GOLD 2019* Global Initiative for Obstructive Lung Disease 2019 Guidelines, *CAT* Chronic Obstructive Pulmonary Disease Assessment Test, *mMRC* Modified Medical Research Council Dyspnoea Scale

Associations of poor adherence to inhalers with COPD outcomes are shown in Table 6. After adjustments, poor adherence was significantly associated with increased probability for having worse health status e.g., CAT **≥**10 [OR (95CI%) 4.86 (1.61–14.69))] and mMRC**≥**2 [OR (95CI%) 2.93 (1.51–5.71)], and having ≥2 exacerbation in the past year [OR (95CI%) 4.68 (1.51–4.44)]. There was also a significant association of poor adherence with GOLD exacerbation status (CAT-based: OR (95CI%) 3.13 (1.49–8.53) and mMRC-based: 3.35 (1.24–9.09), respectively), with patients of poor adherence having a higher probability for becoming stage C or D. Associations of diabetes and poor adherence were independent from each other, however associations with diabetes did not remain significant when occupational status was added into the model.

## Discussion

The current study managed to measure comorbidities and adherence to inhalers, and to convey interesting associations with COPD health status and risk of exacerbations, in a primary care population in rural and semi-urban areas of Greece. A notably high percentage of COPD patients reported poor adherence, with the vast majority being characterized as deliberate non-compliers. Although, much fewer patients presented good-intermediate adherence levels, those were mostly sporadic or unconscious compliers. An overwhelming percentage of COPD patients suffered from more than two comorbidities, with overweight/obesity and hypertension being the two most prevalent. Lastly, COPD outcomes (such as CAT, mMRC, exacerbations and GOLD 2019 classification) found to present significant associations with poor adherence, while comorbidities were not associated with COPD outcomes after adjustments.

### Poor adherence to inhalers

Literature reports good adherence levels ranging from 70 to 90% in clinical trials, but this deteriorates in clinical practice and PHC (i.e. 10–40%) [12, 14, 36]. The findings in this PHC population residing in rural/semi-urban areas of Greece (only 26% with good/intermediate adherence), agree with previously reported rates [14, 36]. This may reflect the inability of physicians to provide inhaler training and educate patients, or the patients’ inability to receive and comply to health information or just the insufficient or incorrect use of the devices [37]. However, it warrants attention the fact that nearly 70% of patients found to have deliberate non-compliance in the present Greek COPD population. This is in line with findings from another Greek rural population in 2014, where 46.4% of COPD and asthma patients had stopped their medications completely, decreased dosages or used similar medications that had in the past, during the austerity period [4], highlighting the impact of the global financial crisis on COPD patients’ adherence to therapy in rural areas. Furthermore, poor adherence to inhalers in our study could be explained by the fact that the majority of the patients were retired and a portion were unemployed, striving to cope with the ongoing financial crisis. In a Greek nationwide COPD study (Greek Obstructive Lung Disease Epidemiology and health ecoNomics: GOLDEN study) [16] which included both urban and semi-urban/rural areas, a slightly higher proportion of subjects reported good adherence, however less subjects were unemployed or retired in that study compared to ours. This observation supports the hypothesis that higher rates of poor adherence in rural areas might be attributed to poorer socioeconomic status, an association indirectly also seen in our study (e.g. occupational status vs. poor adherence).

We found that poor adherence to inhalers is associated with higher risk of exacerbation and worse health status. In a Greek COPD study, omission of medication was also associated with risk of exacerbations [16]. Furthermore, adherence to inhalers has been found to reduce disease severity and related exacerbations [38–40]. In agreement with our findings is also the study by Montes et al. who they also used TAI questionnaire for assessing adherence to inhalers, and found that poor adherence was significantly associated with worse CAT score and higher risk for exacerbations [12]. These findings suggest the importance of adherence to inhalers which may have a direct impact on reducing exacerbations and improving symptoms.

### A focus on comorbidities

As far as comorbidities in COPD patients are a concern, the findings of the current study are in line with EPIC [41] and EPOCA [42] studies that found at least two comorbidities in 39 and 42% of patients, respectively (42.5% in our study). However, in the Greek GOLDEN study 55% of COPD subjects had at least 2 comorbidities [16]. This high prevalence of comorbidities in COPD patients, projects the significance of managing this major health asset that influences overall health status and QoL.

Additionally, our finding of a significant burden of comorbid conditions in individuals with COPD supports previous investigations, with overweight/obesity, hypertension and diabetes being the most frequent [43–45], potentially due to shared underlying risk factors and mechanisms [46]. The large number of overweight/obese COPD patients nearly doubled the proportion of subjects with ≥2 comorbidities, when BMI (≥25 Kg/m^2^) was included in the comorbidity sum score. Prevalence of overweight/obese in our study does not deviate from intercountry data [47]. However, we have not found an association between BMI and GOLD status. There are a few studies assessing the effect of BMI on COPD outcomes, suggesting that obesity, especially if mild-moderate, does not influence COPD symptoms [48, 49].

Moreover, studies suggest that a large proportion of COPD subjects reaching PHC but also secondary/tertiary care frequently have echocardiographical abnormalities proving underlying, sometimes undiagnosed, hypertension and heart disease [50–52]. However, these echocardiographic abnormalities had limited impact on CAT score [52]. Similarly, we showed null associations between CAT and mMRC and heart disease and hypertension.

GERD is one of the most common gastrointestinal conditions in the general population and it is also listed by GOLD as a common comorbidity in COPD [19]. Evidence suggesting that GERD might worsen COPD outcomes remains controversial and the pathophysiological relationship between comorbid GERD and COPD remains unclear [53]. We did not find any association between GERD and COPD outcomes, however it is important to note that only GERD had a significant association with inhaler adherence in our study. Most data come from observational studies thus causal inference is difficult. However, the positive effect of GERD on inhaler adherence could be misleading with regards to which of the two is the cause and which is the outcome. There are studies proposing confounding by indication e.g., more symptoms due to GERD increase need for inhaler and therefore better adherence, while other studies suggest that respiratory medications are possible risk factors for GERD [53, 54], and others propose share risk factors for both diseases e.g. smoking [53]. More studies are needed to understand causal inference.

Data of the German COPD cohort COSYCONET were the first that revealed associations of COPD GOLD status with a number of comorbid conditions [25]. Even though we found an association of diabetes with GOLD status, this association did not remain significant after adjustment for occupational status, neither any of the associations of the rest of the comorbidities. This discrepancy and/or smaller signal may be explained by large difference in characteristics including socioeconomic status, study settings and prevalence of comorbidities with our Greek population, and also by the smaller sample size of this study.

### Clinical implications

According to cumulating evidence in the literature, management of COPD patients may benefit by raising PHC professionals awareness and attention for assessing patient adherence to inhalers as well as for screening and managing comorbidities regularly. The findings of the current study highlight that the presence of poor adherence to inhaled medication influences COPD outcomes. PHC physicians could become the lead coordinators of adherence-oriented interventions or COPD action plans, since they have the closest and continuous relation with COPD and chronic patients. They could help them comprehend the chronic nature of disease and effectiveness of treatment, importance of complete adherence to inhaled therapy and necessity of other supportive actions; including smoking cessation and exercise [36, 48]. Furthermore, treatment of comorbidities should become an important part of COPD management as it may influence inhaler adherence and overall disease status.

### Strengths and limitations

To the best of our knowledge, this is the first Greek study linking poor adherence and comorbidities to COPD outcomes in patients residing in rural and semi-urban settings in a real-life study. Additionally, assessment of type of adherence added value on translating patients’ compliance. The overall characteristics (e.g., male [5] and overweight/obese [47] overrepresentation), number of comorbidities and most common comorbidities of the present COPD population do not deviate much from those in a nationwide COPD study [16, 55]. On the other hand, several limitations should be discussed along with current findings. Primarily, the cross-sectional design may have inserted several biases, while it lacks ability to show causal associations. Patients are representative solely for PHC population of non-urban population, rather than those attending only secondary-tertiary care facilities. Unfortunately, in the Greek national healthcare system spirometry was not available in the rural primary care setting of this study, neither a linkage between information provided by specialists and GPs, therefore associations with outcomes could not be corrected for airflow obstruction at the time of assessment. In addition, in Greece patients can bypass the GP and visit a specialist or hospital without a referral, and since an electronic medical record linking all GPs with specialists and hospitals was not available at the time, misclassification of outcomes (e.g., exacerbations, hospitalizations) could exist.

## Conclusions

Poor adherence to inhalers and comorbidities are both prevalent in COPD patients of primary care residing in rural/semi-urban areas of Greece, with adherence influencing COPD outcomes. Raising awareness of patients and physicians on the importance of comorbidities control and inhaler adherence may lead to interventions and improve outcomes.

## Data Availability

Individual-level data used for this study are held within the servers of the UNLOCK study and the Department of Social Medicine and could not been publicly available due to privacy reasons. Thus, the datasets generated and/or analyzed during the current study are not publicly available due to confidentiality agreement but are available from the corresponding author on reasonable request as long as the request meets the ethics.

## Abbreviations

COPD: Chronic obstructive pulmonary disease
QoL: Quality of life
GOLD: The Global Initiative for Chronic Obstructive Lung Disease
CAT: COPD Assessment Test
mMRC: Modified Medical Research Council Dyspnea Scale
SD: Standard deviation
PHC: Primary health care
EPIC: The Epidemiology and Impact of COPD Asia survey
EPOCA: Enfermedad Pulmonar Obstructiva Crónica en Acción study
COSYCONET: COPD and SYstemic consequences-COmorbidities NETwork
LASSYC: The Latin American Study of 24-hour Symptoms in Chronic Obstructive Pulmonary Disease
GOLDEN: Greek Obstructive Lung Disease Epidemiology and health ecoNomics study
